# Corneal stromal stem cells reduce corneal scarring by mediating neutrophil infiltration after wounding

**DOI:** 10.1371/journal.pone.0171712

**Published:** 2017-03-03

**Authors:** Andrew J. Hertsenberg, Golnar Shojaati, Martha L. Funderburgh, Mary M. Mann, Yiqin Du, James L. Funderburgh

**Affiliations:** Department of Ophthalmology, University of Pittsburgh School of Medicine, Pittsburgh, Pennsylvania, United States; University of Reading, UNITED KINGDOM

## Abstract

Corneal scarring limits vision for millions of individuals worldwide. Corneal transplantation (keratoplasty) is the standard of care for corneal opacity; however, it bears the risk of graft rejection and infection and is not universally available. Stem cell therapy holds promise as an alternative to keratoplasty. Stem cells from human corneal stroma (CSSC) induce regeneration of transparent corneal tissue in a mouse wound-healing model. In this study we investigated the mechanism by which CSSC prevent deposition of fibrotic tissue. Infiltration by CD11b^+^/Ly6G^+^ neutrophils and myeloperoxidase expression were increased in corneas 24 hr after corneal wounding but were reduced in CSSC-treated wounds. Secretion of TSG-6, a protein known to regulate neutrophil migration, was up-regulated in CSSC in response to TNFα and as CSSC differentiate to keratocytes. In vivo, wounded mouse corneas treated with CSSC contained human TSG-6. Inhibition of neutrophil infiltration into cornea by CSSC was reversed when TSG-6 expression was knocked down using siRNA. Silencing of TSG-6 expression in CSSC reduced their ability to block scarring and the expression of mRNA for fibrosis-associated proteins collagen III, tenascin C, and smooth muscle actin in wounded corneas. Neutropenic mice exhibited a significant reduction in corneal scarring and fibrotic mRNA expression 2 weeks after wounding. These results support the conclusion that neutrophil infiltration is an essential event in the fibrotic response to corneal damage and that prevention of scarring by CSSC is mediated by secretion of TSG-6 by these cells.

## Introduction

Corneal blindness resulting from ocular trauma or infection affects 7–10 million people worldwide[1]. Currently the only treatment option for most of these individuals consists of corneal transplantation (lamellar or penetrating keratoplasty), a procedure complicated by tissue rejection and limited by the supply of donor tissue[2]. Consequently, there is an increasingly important need to develop alternative therapies for these patients.

Alternatives to corneal transplantation including prostheses, cell therapy, and bioengineered tissues are currently being studied with the hope of becoming the standard of care for treatment of corneal scars. Indeed, collagen-based engineered tissue has been successfully employed as partial thickness corneal grafts in animals, and is currently in human clinical trials [3–5]. Stem cells are also being investigated for use in cell therapy as well as for engineering of biosynthetic corneal tissue. Human corneal stromal stem cells (CSSC) are of particular interest for these applications as they represent the natural progenitors for keratocytes, cells that make up the corneal stroma. CSSC isolated from human limbal stromal tissue have been shown to restore transparency in a genetic model of corneal haze in mice [6–8]. These same cells have also been used to generate organized, collagenous matrices which mimic corneal tissue. This matrix may be useful as bioengineered tissue for transplant[9–11]. More recently, we have shown that limbal biopsy-derived CSSC prevent fibrotic wound healing and promote regeneration of transparent native corneal tissue in a mouse model of corneal wounding [12]. This finding could lead to the use of autologously isolated CSSC to repair damaged corneal tissue in an approach that simplifies the surgical procedure and obviates need for donor tissue. A key to moving forward with CSSC cell therapy is to elucidate the mechanism by which these cells prevent fibrosis and scarring.

Stem cells offer the ability to regenerate damaged tissue, restoring both function and integrity. A number of studies have revealed these characteristics using multiple wound models[13–16]. It is becoming increasingly apparent that immunomodulation by stem cells is important for their anti-fibrotic/pro-regenerative wound healing properties [17–19]. Although several secreted molecules have been investigated for immunosuppressive properties, notable among them is tumor necrosis factor α stimulated gene 6 protein (TSG-6) [20–22]. TSG-6 protein is a matrikine that binds hyaluronan and other glycosaminoglycans. It is expressed by several cell types in response to inflammation[23]. TSG-6 directly inhibits neutrophil migration by binding the chemokine Interleukin-8, a neutrophil chemotactic factor[24]. TSG-6 protein, either applied topically or secreted by bone marrow mesenchymal stem cells, modulates acute-phase inflammation in corneas damaged with ethanol[25–27]. As neutrophils are the first responders to wound sites and serve to recruit other inflammatory cells, it seems likely that preventing neutrophil infiltration at the site of injury may mediate functions of cells present in the wound at later stages of healing.

In the present study, we investigated the mechanism by which CSSC derived from limbal biopsy tissue prevent fibrosis in a mouse model of corneal wounding, using a model described in previous studies[12, 28]. We report that CSSC prevented infiltration of neutrophils after a stromal debridement wound in a TSG-6-dependent manner and that the absence of neutrophils reduced or eliminated corneal scarring and fibrosis.

## Materials and methods

### Limbal biopsy and cell culture

Human corneo-scleral rims, approved for research purposes, from de-identified donors younger than 60 years, were obtained from the Center for Organ Recovery and Education, Pittsburgh PA (www.core.org). Tissue was used within 5 days of enucleation. Research followed the tenets of the Declaration of Helsinki and was approved by the University of Pittsburgh Institutional Review Board (IRB) and CORID (Committee for Oversight of Research and Clinical Training Involving Decedents), Protocol #161. Dissection of limbal stroma was performed as described previously[12]. Briefly, rims were rinsed in Dulbecco’s modified Eagle’s/Ham’s F12 (1:1) medium (DMEM/F12) with antibiotics, and an annular ring of tissue consisting of the superficial limbal epithelium and stroma, 0.5 mm into the cornea, was excised using Vannas scissors while stabilizing the tissue by holding the sclera and conjunctiva with toothed forceps.

Excised limbal segments were incubated in collagenase (0.5 mg/ml) (Sigma-Aldrich, type L) overnight at 37°C. Digests were triturated, incubated for an additional hour, and filtered through a 70 μm, nylon filter to obtain a single-cell suspension. Cells obtained from each segment were seeded into a 25 cm^2^ tissue culture flask in stem cell growth medium[6] (SGM) containing 2% (v/v) pooled human serum (Innovative Research). Culture medium was changed at 3-day intervals, and cells were passaged by brief digestion with TrypLE Express (Life Technologies) when 80% confluent into a 175-cm^2^ T-flask and cryopreserved at passage 1. Cells were used at passage 2 or 3. In expression studies, CSSC were cultured in SGM or in keratocyte differentiation medium (KDM) composed of Advanced DMEM (containing GlutaMAX, Gibco, 12491), ascorbate-2-phosphate (1 mM), fibroblast growth factor–2 (10 ng/ml), and transforming growth factor–β3 (0.1 ng/ml)[9].

### siRNA knockdown of TSG-6 expression

10^6^ CSSC cells grown overnight in a 100 mm culture dish were transfected with TSG-6 siRNA (Santa Cruz Biotechnologies, sc-39819) or a scrambled control siRNA (sc-3007), 42 nM in 9 ml SGM medium using Viromer Blue transfection agent (OriGene Technologies) according to the manufacturer’s instructions. Cells were cultured at 37°C for 24–72 hr in the presence of TNFα (20 ng/ml) to induce TSG-6 expression. Alternately, transfection was continued for 48 hr in SGM before CSSC were used to treat corneal wounds. TSG-6 protein concentration in culture media was assessed using ELISA as previously described [29].

### Corneal wounding

This study was carried out in strict accordance with the recommendations in the Guide for the Care and Use of Laboratory Animals of the National Institutes of Health and The Association for Research in Vision and Ophthalmology Statement for the Use of Animals in Ophthalmic and Vision Research. It was approved by the Institutional Animal Care and Use Committee of the University of Pittsburgh, Protocol # 15025426. Procedures were adopted to minimize pain and suffering in the animal subjects.

Female C57/Bl6 mice, 7–8 weeks of age, were obtained from Charles River Laboratories International Inc., housed in an AALAC-approved ABSL2 facility, and provided an unrestricted standard diet. Groups of 5 mice were anesthetized by intraperitoneal injection of ketamine (50 mg/kg) and xylazine (5 mg/kg). Our previous study and power analysis determined that at least six eyes were required for statistical significance in visible scar analysis and that 2 weeks provided appropriate time points for analysis of gene expression and fibrosis[12, 28]. One drop of proparacaine hydrochloride (0.5%) was added to each eye before debridement for topical anesthesia. Debridement procedures were done as previously described [12, 28] Corneal epithelial debridement was performed by passing an AlgerBrush II (The Alger Company) over the central 2 mm of the mouse cornea. Once the epithelium was removed, a second application of the AlgerBrush II was used, this time applying more pressure to remove the basement membrane and 10 to 15 μm of anterior stromal tissue. Immediately after the procedure mice received ketoprofen (3 mg/kg) for analgesia. Both eyes received the same wounding and treatment.

### Fibrin gel and CSSC application

CSSC were suspended in PBS at 5 × 10^7^ /ml and mixed 1:1 with human fibrinogen (Sigma), 70 mg/ml in PBS and maintained on ice. This concentration was determined to be the maximum number of cells that was retained on the corneal surface during healing. After wounding, 0.5 μl of thrombin (100 U/ml, Sigma) was added to the wound bed, followed immediately by 1 μl of fibrinogen (with or without CSSC). Fibrin gelled in 1 to 2 min, and a second round of thrombin and fibrinogen was applied. The wound was treated with a drop of gentamicin ophthalmic solution (0.3%). The corneal epithelium reformed over the wound in 24 to 36 hours. Eyes were examined daily for signs of rejection and infection for 1 week and weekly thereafter.

### Assessment of scarring

Two weeks after the corneal debridement all eyes were collected and the whole globes were imaged using a dissecting microscope with indirect illumination. Images of the scars were captured and scar area was determined on images of the eyes, with identity of the samples masked, using the Fiji open-source image analysis software package (https://fiji.sc/). Statistical analysis of the values was performed with Prism 7 (GraphPad Prism) using t-tests or ANOVA as noted in the text.

### Induced neutropenia

Neutrophils were ablated in three female C57/Bl6 mice, 8 weeks of age, 24 hours prior to debridement by i.p. injection of 0.5 mg, anti-Ly6G monoclonal antibody 1A8[30]. A second dose was given at the time of debridement. Corneas were wounded as described above without fibrinogen or cellular treatment.

### Quantification of neutrophil infiltration

24 hours after wounding, mice were sacrificed and eyes enucleated. Dissected corneas were cut into quarters and digested in Collagenase Type 1 (Sigma) 820 U/mL in DMEM/F12 + 10% fetal bovine serum at 37°C for 1 hour, vortexing every 20 minutes. Digests were triturated until tissue was completely broken up, and digestion continued for an additional 20 minutes. Cell suspensions were filtered through a 70 μm nylon mesh and pelleted at 340 x g for 10 minutes. Cells were stained in 50 μl staining buffer (phosphate buffered saline, 1% fetal bovine serum) by addition of Fc-Block, anti-CD45-PerCP, anti-Gr1-PE (clone RB6-8C5), and anti-CD11B-AF647 (all BD Biosciences) at 1:50 for an incubation of 30 minutes on ice in the dark. Stained cells pooled from 6 corneas were washed by centrifugation from staining buffer and fixed in 300 ul 1% paraformaldehyde in PBS before analysis by flow cytometry on a FACS Aria III Flow Cytometer (BD Biosciences). For determining cell counts in individual corneas, each sample was spiked with 20,000 fluorescent counting beads (CountBright absolute counting beads; Invitrogen). and the bead count was used to calculate absolute numbers of immune cells per cornea[31].

### Neutrophil myeloperoxidase (MPO) assay

Mouse corneas were excised and dissected 24 hr after wounding, removing all residual iris and scleral tissues, and each cornea was incised radially. Individual corneas were placed in 0.3 ml tissue extraction buffer (ThermoFisher- Life Technologies) containing 1:100 protease inhibitor cocktail (Sigma P8340) and disrupted by sonication in 4 x 30-second bursts, with cooling on ice between bursts. The homogenate was centrifuged for 15 min at 14,000 x g at 4°C. MPO activity was determined in 1:20 dilution of the homogenate using a fluorometric immunoassay assay (R & D Systems, DY3667) according to the manufacturer. Each sample was analyzed in triplicate and MPO was calculated from a standard curve.

### Quantitative real time reverse transcription PCR (qPCR)

6 corneas per group were dissected and pooled in 700 μl RLT extraction reagent (Qiagen) and disrupted with MagNA Lyser green beads using 6 cycles @ 6,000 RPM with intermittent cooling in a MagNA Lyser Instrument (Roche). The extracts were centrifuged briefly to collect lysate, and further processed using Qiashredder (Qiagen). RNA was isolated by Qiagen RNeasy Miniprep and 500 ng total RNA was transcribed to cDNA using SuperScript III (Life Technologies) as previously described[12]. cDNA and target primers (Table 1) were combined with SYBR Green Real-Time Master Mix (Life Technologies) and real-time polymerase chain reaction run and data analyzed using the StepOnePlus Real-Time PCR System (Applied Biosystems) [12]. Relative mRNA abundance was compared by ΔΔCt method using 18S RNA as in internal control[12].

**Table 1:** PCR primers.

Gene	Protein	Accession	Primers
*Acta2*	smooth muscle actin	NM_007392.3	F: TGTGCTGGACTCTGGAGATG
			R: GAAGGAATAGCCACGCTCAG
*Col3A1*	collagen type III	NM_009930.2	F: CGTAAGCACTGGTGGACAGA
			R: CGGCTGGAAAGAAGTCTGAG
*Tenc*	tenascin C	NM_011607.3	F: GACTGCCCTGGGAACTGTAA
			R: CATAGCCTTCGAAGCACACA
*TNFAIP6*	TSG-6	NM_007115.3	F: AAGCACGGTCTGGCAAATACAAGC
			R: ATCCATCCAGCAGCACAGACATGA

### Immunostaining

Immunostaining of mouse tissue was performed on 8 μm cryostat sections post-fixed in ice-cold 4% paraformaldehyde, for 10 minutes and blocked with 10% heat-inactivated goat or donkey serum in phosphate-buffered saline (PBS). An antibody specific for human TSG-6 (1:150, Santa Cruz Biotechnology) was incubated on the sections at 4°C overnight. Slides were washed three times in PBS and stained with AlexaFluor 546–conjugated anti-rat secondary antibody (Life Technologies) at 1:1000 for 2 hours at room temperature. Slides were subsequently washed three times in PBS before staining with DAPI for 15 minutes at room temperature. Slides were imaged with an Olympus FluoView 1000 confocal microscope with a 20X oil objective.

## Results

### TSG-6 expression by CSSC

**Fig 1:**
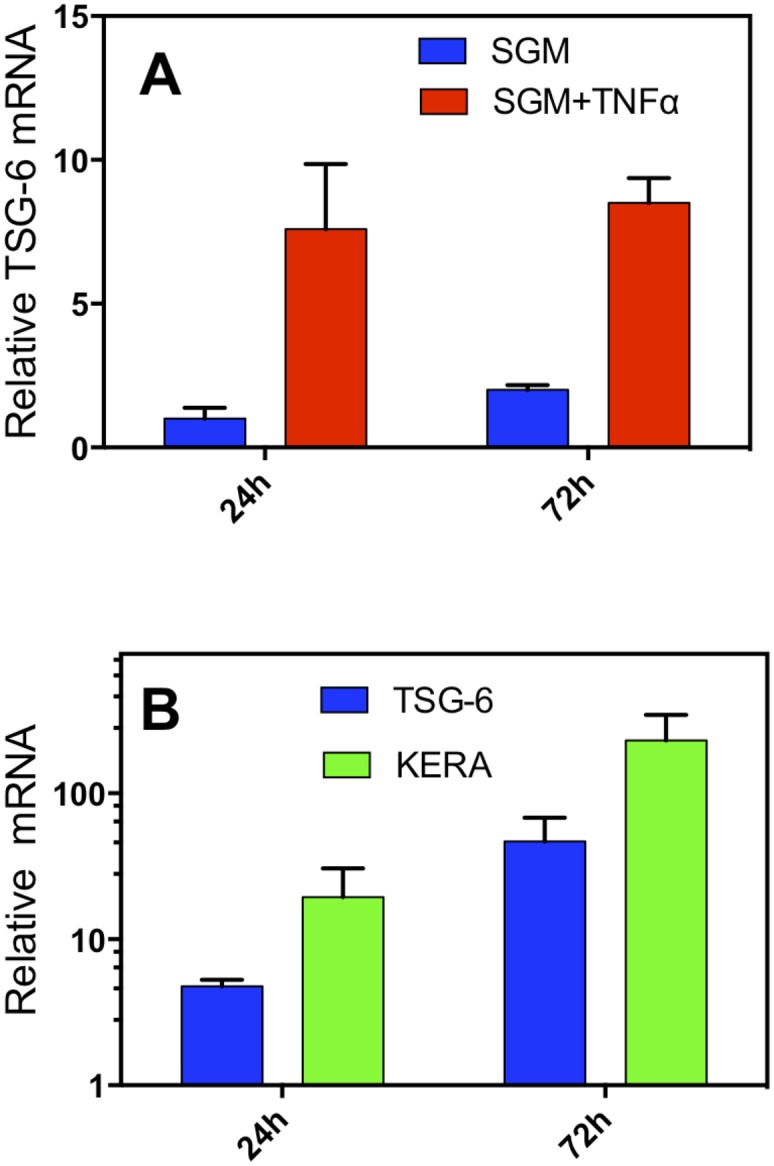
TSG-6 mRNA is expressed by activated CSSC. (**A**) CSSC were exposed in culture to TNFα 10 ng/ml, IFN-γ 25 ng/ml (SGM-TNFα) in stem cell growth medium (SGM) for 24 and 72 hr and mRNA for TSG-6 was detected by qPCR as described in Materials and methods. (**B**) CSSC were switched into keratocyte differentiation medium for 24 and 72 hr. Expression of mRNA for *TNFAIP6* (TSG-6) and *KERA* (keratocan) was determined by qPCR. mRNA levels were normalized to that of untreated cells which was arbitrarily set to equal 1. Expression levels for both genes were significantly greater than those in unstimulated cells at both time points (S1 Tables).

TSG-6 is a hyaluronan-binding protein induced by tumor necrosis factor alpha (TNFα) in several different cell types including bone marrow-mesenchymal stem cells[32]. TSG-6 expression by CSSC has not been previously documented. After treatment of CSSC with TNFα, TSG-6 mRNA for was increased about 9-fold compared to untreated cells at both 24 and 72 hr (Fig 1A, S1 Tables). TSG-6 mRNA expression was also stimulated as CSSC differentiated to keratocytes. After 72 hours in differentiation medium, expression of mRNA for TSG-6 was upregulated nearly 50-fold compared to undifferentiated cells (p<0.001) (Fig 1B, S1 Tables). Keratocan is a keratocyte-specific protein upregulated as CSSC differentiate[33, 34]. Keratocan mRNA expression was upregulated in a similar fashion to that of TSG-6 mRNA in differentiating CSSC (Fig 1B). CSSC therefore express TSG-6 in response to an inflammatory environment as well as during the process of differentiation into keratocytes.

**Fig 2:**
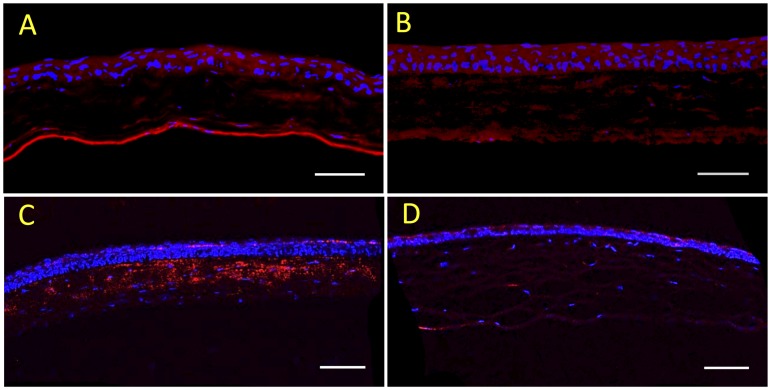
TSG-6 accumulates in CSSC-treated wounds. Mouse corneas were treated immediately after a debridement wound with (**A-C**) CSSC in fibrinogen or (**D**) fibrinogen only as described under Materials and Methods. Corneas were fixed and cryosections were stained at (**A**) 2 days, **(B)** 14 days, and (**C, D**) 4 weeks after wounding using antibody specific for human TSG-6 (red) and DAPI to stain cell nuclei (blue).

Previously we showed CSSC to prevent fibrosis in mouse corneal wounds by engrafting CSSC into the stroma in fibrin gel after debridement of the epithelium and the corneal basement membrane[12]. To examine whether CSSC actively secrete TSGG-6 into these wounds we immunostained the tissue with an antibody against human TSG-6. Wounds with stem cells at 2 and 14 days of treatment showed weak stromal staining for human TSG-6 (Fig 2A and 2B) however by four weeks after wounding, TSG-6 protein was clearly localized in the anterior stroma (Fig 2C). The immunostaining used human-specific antibody, supporting the idea that TSG-6 protein originated from the engrafted CSSC. Eyes wounded without CSSC did not stain for human TSG-6 (Fig 2D).

### CSSC reduce neutrophil infiltration after corneal wounding

**Fig 3:**
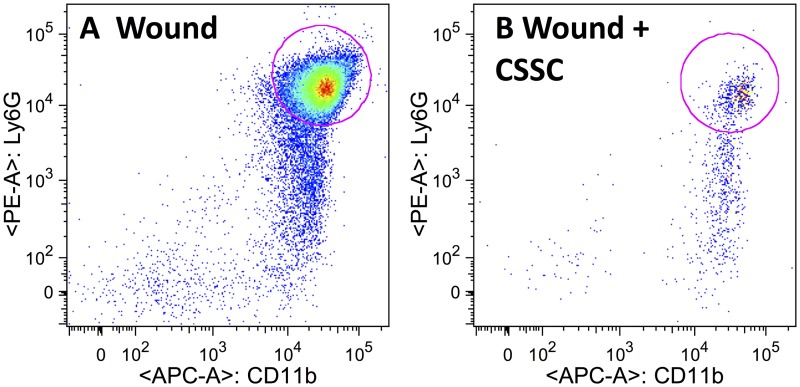
CSSC treatment reduces corneal neutrophil filtration after wounding. Cells pooled from 6 corneas were isolated 24 hr after wounding and separated by flow cytometry as described under Materials and Methods using antibodies to antigens present on the surface of neutrophils: CD45, Ly6G and CD11b. (**A**) Cells from wounds treated with fibrinogen only. (**B**) Cells from wounds treated with CSSC in fibrinogen. The total number of immune cells (CD45^+^) as well as the number of neutrophils (Ly6G^+^/CD11b^+^ cells) is provided in Table A of S2 Tables. Cell numbers per cornea in a similar experiment are shown in Table B of S2 Tables.

Corneal wounding results in a rapid infiltration of neutrophils in response to mechanically-induced tissue damage[35, 36]. Using flow cytometry, we identified a population of CD11b^+/^ Ly6G^+^ cells in corneal digests at 24 hr after wounding (Fig 3A). The application of CSSC onto the denuded stroma immediately after wounding resulted in a strong reduction in stromal neutrophils after the same time-period (Fig 3B) (Table A in S2 Tables). Fig 3 used cells pooled from multiple corneas. When the analysis was carried out on single corneas, the reduction of neutrophils per cornea was found to be >80% (p< 0.05) and the proportion of neutrophils as a component of total inflammatory cells (CD45^+^) was also reduced by 70% (p< 0.05) (Table B in S2 Tables). This reduction suggests that the CSSC exert an anti-inflammatory effect that is neutrophil-specific.

### CSSC reduce corneal neutrophil infiltration via TSG-6

**Fig 4:**
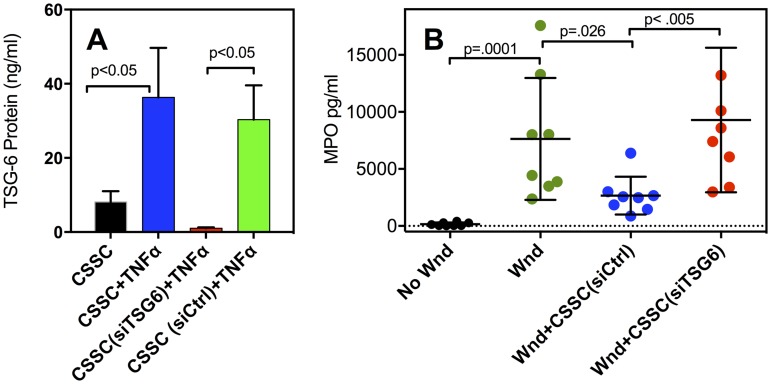
Knockdown of TSG-6 restores neutrophil infiltration after wounding. Graph **(A)** shows TSG-6 in culture media detected by ELISA after 72 hr culture in stem cell growth media (SGM). Untreated CSSC, (black); CSSC cultured in 20 ng/ml TNFα and 10 ng/ml IFN-γ, (blue); CSSC transfected with siRNA against TSG-6 mRNA and incubated in TNFα+IFN-γ, (red); CSSC transfected with a scrambled siRNA and incubated in TNFα+ IFN-γ, (green). Error bars show standard deviation (SD) of quadruplicate assays (see Table A of S3 Tables). Graph (**B**) shows MPO assay of extracts of individual corneas as described in Materials and Methods. Corneas were: non-wounded (No-Wnd, black); wounded-untreated (Wnd, green); wounded and treated with CSSC (CSSC-siCtrl, blue); wounded and treated with CSSC with TSG-6 knocked down (CSSC-siTSG6, red). Error bars show S.D. and p values from t-tests comparing individual pairs of samples as described in Table B of S3 Tables.

TSG-6 has recently been shown *in vitro* to inhibit neutrophil migration via its interaction with Interleukin 8 [24]. The role of TSG-6 in the suppression of corneal neutrophil infiltration was tested by knockdown of TSG-6 mRNA using siRNA. Secretion of TSG-6 protein into culture media in response to TNFα was found to be >95% suppressed in CSSC treated with TSG-6 siRNA (Fig 4A) (p<0.004) (Table A in S3 Tables) whereas scrambled siRNA (siCTRL) had no significant effect (Fig 4A).

The role of TSG-6 as a mediator of neutrophil infiltration in vivo was examined using CSSC in which TSG-6 secretion was knocked down. Quantitative analysis of neutrophil infiltration was assessed by measuring MPO, a protein highly expressed in neutrophils, in extracts of individual corneas. As shown in Fig 4B (black circles), MPO was not detected in un-wounded corneas but was elevated at 24 hr after wounding (Fig 4B, green). In wounds treated with CSSC expressing TSG-6, wounded corneas contained significantly less MPO at 24 hr (Fig 4B, blue), a value not statistically different from unwounded controls (p = 0.25). However, in wounds treated with CSSC in which TSG-6 secretion was knocked down (Fig 4B, red), corneas contained MPO at the same level as untreated wounds. Suppression of neutrophil infiltration is therefore directly correlated to secretion of TSG-6 by CSSC. Statistical analysis of the data is available in Table A and B of S3 Tables.

### CSSC TSG-6 knockdown restores corneal fibrosis after wounding

**Fig 5:**
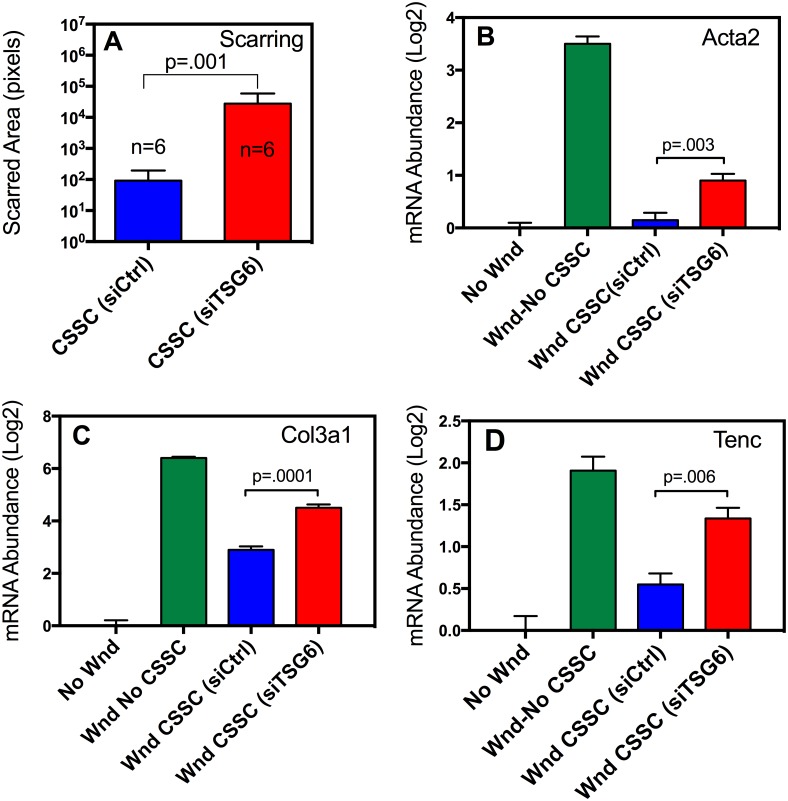
Knockdown of CSSC TSG-6 expression increases scarring and fibrosis after wounding. (**A**) Scarred area was measured in individual eyes (n = 6) as described in Materials and Methods (images provided in S1 Fig and analysis in Table A of S4 Tables). Eyes were treated with CSSC transfected with siRNA against TSG-6 (CSSC-siTSG6, blue) or with a scrambled RNA (CSSC-siCtrl, red). Relative expression of fibrotic genes was measured by qPCR for (**B**) Act2a; (**C**) Col3a1; (**D**) Tnc. Corneas were: not wounded, (No Wound, black); wounded and untreated (Wound-No CSSC, green); wounded and treated with CSSC (CSSC-siCtrl, blue); wounded and treated with CSSC with TSG-6 knocked down (CSSC-siTSG-6, red). Values were normalized, setting non-wounded corneas = 0. Error bars are S.D. of triplicates. p-values are derived from t-tests of pairs of data. (Table B in S4 Tables).

The clinical outcome of corneal wounding is vision-impairing scarring due to corneal fibrosis. When fibrotic matrix replaces the specialized connective tissue present in corneal stroma, light is scattered resulting in vision impairment [37]. We assessed corneal fibrosis by measuring the area of visible scars and by assessing genes associated with fibrosis using qPCR at 14 days post wounding[12, 28]. Corneas treated by CSSC with TSG-6 knocked down developed scars of significantly greater area compared to corneas treated with scrambled siRNA (Fig 5A and S1 Fig). qPCR analysis of the RNA from these corneas showed increased mRNA expression for smooth muscle actin (Fig 5B), Collagen III (Fig 5C), and Tenascin C (Fig 5C). Wounded corneas treated with CSSC exhibited reduced expression of these mRNAs; however, knockdown of TSG-6 resulted in significantly increased expression of these genes compared with CSSC expressing TSG-6. These results support the idea that mediation of scarring and fibrosis by CSSC is related to the ability of these cells to suppress neutrophil infiltration after wounding (Fig 5B–5D).

### Neutropenia prevents fibrosis after wounding

**Fig 6:**
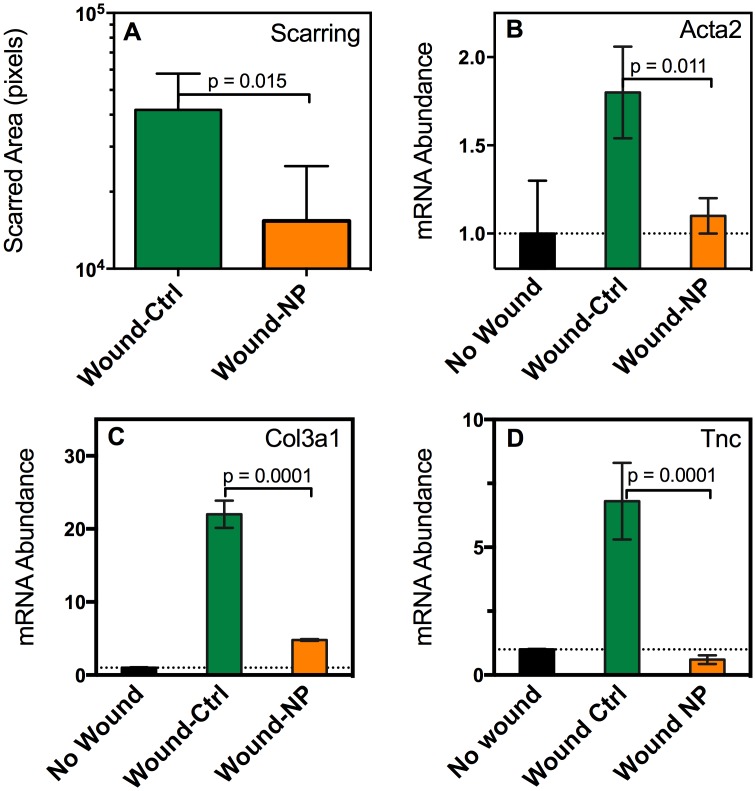
Neutropenic mice exhibit reduced corneal fibrosis. Corneal wounds were carried out in untreated mice (Wound-Ctrl, green), and in mice made neutropenic by injection of anti-Ly6G antibody, as described in Materials and methods (Wound NP, orange). (**A**) Scar area (n = 6) and expression for fibrotic genes (**B,C,D**) were assessed at 2 weeks as described in Fig 5. Images of scarred corneas are provided in S2 Fig and statistical analysis is shown in S5 Tables.

Neutrophil infiltration is blocked by the CSSC secretion of TSG-6; however, a direct role of neutrophils in corneal scarring has not been established. Corneal wounding consequently was carried out in mice in which a neutralizing antibody to Ly6G (clone 1A8) was injected to induce acute neutropenia [38–41]. Images of the corneal scarring are available in S2 Fig. The area of corneal scarring at 14 days was significantly reduced in the wounded neutropenic mice (Fig 6A) compared with the area of scars in control mice. The neutropenic mice also exhibited significantly reduced expression of mRNA for smooth muscle actin, collagen III, and tenascin C (Fig 6B–6D) compared to that of corneas from wounded control mice. These data support the hypothesis that neutrophil involvement in corneal wounds is indeed important to the clinical outcome associated with corneal scarring.

## Discussion

Previously, a study from this laboratory showed that CSSC in fibrin gel engraft into the mouse cornea and prevent corneal scarring in response to corneal debridement wounds[12]. The current experiments were designed to elucidate the mechanism of this phenomenon. We found that CSSC engrafted into the wound reduced the number of neutrophils present in the cornea 24 hr after wounding. TSG-6, a protein known to prevent neutrophil migration, was secreted by the engrafted CSSC and remained in the anterior stroma for at least four weeks. Knockdown of TSG-6 expression in CSSC restored neutrophil infiltration into the wounded corneas and reduced the ability of CSSC to prevent scarring. Finally, we observed that neutropenic mice exhibited reduced scarring and fibrosis compared to untreated mice. The sum of these observations argues that alteration of corneal neutrophil infiltration by CSSC plays a key role in mediating the cellular events resulting in formation of stromal scar tissue. Such a role for neutrophils has not been previously documented in corneal wound healing, nor is it well established as a mechanism active in wound healing in other tissues such as dermis.

The mechanism by which CSSC control neutrophil infiltration appears to be secretion of TSG-6. This is consistent with reports that TSG-6 protein suppresses human neutrophil migration by interaction with the chemokine CXCL8 [24]. Mice lack CXCL8 but TSG-6 also exhibits anti-inflammatory properties in mice, suppressing corneal inflammation after chemical damage to the epithelium [26]. TSG-6 is a major contributor to the anti-inflammatory functions of bone marrow-derived mesenchymal stem cells BM-MSC [42]. CSSC are categorized as mesenchymal stem cells and share a number of properties with BM-MSC. In this study we observed that, like BM-MSC, CSSC respond to TNFα exposure with a strong upregulation of TSG-6 mRNA and protein. Interestingly, culture media containing FGF-2 and TGF-ß3, which induce keratocyte differentiation, also upregulated TSG-6 in CSSC (Fig 1). When CSSC are engrafted into a healing wound they differentiate to keratocytes [12], and in this study we found them to secrete TSG-6 protein into the anterior stroma (Fig 4). Our previous analysis of gene expression in CSSC and keratocytes [8] found that keratocytes expressed higher levels of TNFAIP6 (TSG-6 mRNA) than CSSC or corneal fibroblasts (S6 Tables). This suggests that TSG-6 may be a low-level component of normal corneal stroma, contributing to the anti-inflammatory properties of this tissue. Knockdown of mRNA for TSG-6 in CSSC blocked their ability to inhibit neutrophil infiltration and also allowed the formation of visible scars and the upregulation of mRNA for fibrotic markers: smooth muscle actin, collagen type III, and tenascin c (Fig 5). The linkage of corneal neutrophil infiltration after wounding with subsequent scarring was confirmed in mice with neutropenia induced by injection of anti-Ly6 antibody. Neutropenic mice had greatly reduced visible scarring and fibrotic gene expression similar to unwounded controls. These results support the idea that CSSC exert their anti-fibrotic activity primarily by reducing infiltration of neutrophils, an early inflammatory response to traumatic wounding.

Cellular events in healing corneal wounds follow a temporal program typical for many other tissues. Cells near the wound site undergo apoptosis and neutrophil infiltration begins within 6 hr, peaking around 24 hr [43]. Few neutrophils remain at 72 hr. Activated tissue fibroblasts migrate into the wound site beginning 2 days after wounding and differentiation of fibroblasts to myofibroblasts begins at 3–5 days after wounding [44, 45]. Expression of fibrosis-related genes is detected 2 weeks after wounding and deposition of scar tissue occurs 14–28 days after wounding [28]. This chronology shows a significant time gap between the presence of neutrophils in the wound and deposition of scar tissue, suggesting an indirect, rather than direct, effect of the neutrophils on the fibrotic process.

Myofibroblast differentiation is a key step in the generation of corneal scars and occurs as a response of fibroblasts to TGFß [46](review). Macrophages have been proposed as a source of TGFß in wounds in several tissues [47, 48]. These myeloid cells appear within 2 days after trauma but secrete TGF-ß1 during a later, resolution phase of healing following the appearance of myofibroblasts in the cornea [46]. Several studies support the idea that migrating corneal epithelial cells are essential for the presence of myofibroblasts in healing corneal wounds and that epithelial cells represent the source of TGFß in the healing cornea[49–52]. In neutropenic mice, corneal migration after wounding is greatly reduced and wound closure is delayed[43, 53]. The implication of these two findings suggest that dysregulation of epithelial migration as a result of altered neutrophil infiltration could alter the availability of TGFß required for myofibroblast formation and scarring.

Additional effects of neutrophils may result from secretory products left behind in the wound bed by these cells. Activated neutrophils secrete several enzymes, including neutrophil elastase, a protease recently shown to induce differentiation of fibroblasts to myofibroblasts in lung tissue [54]. Neutrophil elastase is incorporated into fibrous DNA-containing structures known as neutrophil extracellular traps (NETs) which remain in tissue at least 5 days after wounds [55]. NETs localize and kill bacteria but also are found in sterile inflammation and healing wounds[56]. NETs are formed in response to a number of factors encountered in corneal wounds, including TNFα and IFN-γ[56]. NETs, like elastase, can induce myofibroblast differentiation[57]. Neutrophils also secrete fibronectin, a matrix protein absent in normal stroma, which is required for myofibroblast differentiation[58]. These recently described properties of neutrophils are consistent with a model in which fibroblasts migrate into the wound and differentiate to myofibroblasts under the influence of TGF-ß2 secreted by epithelium and neutrophil fibronectin and elastase in NETs in the wound area. Reduction in neutrophils would alter the kinetics of TGF-ß2 secretion and the abundance of the NETs, thereby reducing the number of myofibroblasts and the secretion of fibrotic matrix.

The role of neutrophils in fibrosis is a novel concept and testing of the model proposed above may provide insight into the cellular mechanisms of corneal scarring. TSG-6, while important, may not represent the sole component effecting tissue regeneration by CSSC. As seen in Fig 5, CSSC without TSG-6 expression maintain some ability to block fibrotic gene expression. Exploration of this mechanism may be clinically relevant. Only a limited proportion of individuals suffering corneal wounds will be able to obtain medical care in the brief period of time during which neutrophils populate the cornea. It is therefore important to explore the mechanism by which CSSC resolve corneal opacity in a post-inflammatory environment[6]. Understanding and enhancing this regenerative character of CSSC will allow treatment of the millions of individuals who have existing corneal opacities but no access to corneal keratoplasty.

## Supporting information

S1 Tables Data supporting Fig 1. (PDF)

S2 Tables Statistical data supporting Fig 3. (PDF)

S3 Tables Statistical data supporting Fig 4. (PDF)

S4 Tables Data supporting Fig 5. (PDF)

S5 Tables Supporting data for results displayed in Fig 6. (PDF)

S6 Tables Expression levels of KERA and TNAIP6 in gene array. (PDF)

S1 Fig Scarring in eyes treated with CSSC after TSG-6 knock-down. (PDF)

S2 Fig Scarring in eyes of neutropenic mice. (PDF)
