# Supplementary material for: Corneal stromal stem cells reduce corneal scarring by mediating neutrophil infiltration after wounding
Source: PLoS One. 2017 Mar 3;12(3):e0171712. doi: 10.1371/journal.pone.0171712 (PMC5336198; doi:10.1371/journal.pone.0171712)
Supplement: S1 Tables — (PDF) [file pone.0171712.s001.pdf]

S1 Tables. Data supporting Fig 1.

Table A. TSG-6 mRNA Up-Regulation in Response to TNF-alpha*

Culture Conditions	Relative TSG6 mRNA**	S.D.	n	p value
SGM (24 hr)	1	0.38	3	
SGM (72 hr)	2	0.17	3	0.6432
TNFα (24 hr)	7.6	2.25	3	0.0004
TNFα (72 hr)	8.5	0.87	3	0.0002

* Evaluation by ordinary one-way ANOVA with Dunnett's multiple comparisons test.

** Values are calculated relative to CSSC in SGM (24 hr).

TableB. mRNA Up-Regulation in Keratocyte Differentiation Medium*

Culture Conditions	Relative mRNA**	S.D.	n	p value
	**TSG-6**			
SGM (24 hr)	1.0	0.1	3	
KDM (24 hr)	4.8	0.5	3	0.9718
KDM (72 hr)	46.7	21.3	3	0.0071
	**Keratocan**			
SGM (24 hr)	1.0	5.2	3	
KDM (24 hr)	19.5	11.1	3	0.0028
KDM (72 hr)	229.9	114.3	3	0.0286

* Evaluation by ordinary one-way ANOVA with Dunnett's multiple comparisons test.

** Values Calculated relative to SGM (24 hr) for each gene.
