# Supplementary material for: Corneal stromal stem cells reduce corneal scarring by mediating neutrophil infiltration after wounding
Source: PLoS One. 2017 Mar 3;12(3):e0171712. doi: 10.1371/journal.pone.0171712 (PMC5336198; doi:10.1371/journal.pone.0171712)
Supplement: S2 Tables — (PDF) [file pone.0171712.s002.pdf]

S2 Tables. Statistical data supporting Fig 3

Table A Immune Cells in Mouse Corneal Wounds at 24 hr*

	CD45	Neutrophil	Neutrophil %
**Wound**	31424	27089	86
**Wound+CSSC**	1196	583	49

* Data from Fig 3.

Table B Flow analysis of Neutrophils in Single Wounded Corneas*

	CD45	Neutrophil	Neutrophil %
Wound Control	413	89	21.5
Wound Control	262	25	9.54
Wound Control	344	84	24.4
Wound Control	420	14	3.33
Wound+CSSC	225	15	6.67
Wound+CSSC	341	4	1.17
Wound+CSSC	299	14	4.68
Wound+CSSC	183	1	0.55
	**Mean**		
**Wound Control**	359.8	53.0	14.7
**Wound+CSSC**	262.0	8.5	3.3
	**SD**		
**Wound Control**	73.6	39.0	9.9
**Wound+CSSC**	71.2	7.0	2.9
	**p value****		
	0.0525	0.033	0.0347

* Cell count per cornea as described in Methods. Data not presented in manuscript.

** Determined from unpaired, single-tailed t-test.
