# Supplementary material for: Corneal stromal stem cells reduce corneal scarring by mediating neutrophil infiltration after wounding
Source: PLoS One. 2017 Mar 3;12(3):e0171712. doi: 10.1371/journal.pone.0171712 (PMC5336198; doi:10.1371/journal.pone.0171712)
Supplement: S3 Tables — (PDF) [file pone.0171712.s003.pdf]

S3 Tables. Statistical data supporting Fig 4

Table A ELISA for TSG6- Protein*

	TSG-6 (ng/ml) mean	SD	n	p value
**Untreated CSSC**	8.1	5.0	3	0.0421
**CSSC+TNFa**	36.3	23.1	3	
**CSSC(siTSG6)+TNFa**	1.0	0.5	3	0.0363
**CSSC (siCtrl)+TNFa**	30.3	16.0	3	

* As summarized in Fig 4A in manuscript.
** p values calculated from piarwise comparison of all samples in ordinary one-way ANOVA with Fishers LSD test.

Table B ELISA for MPO*

	MPO Concentration (pg/ml)			
	No Wound	Wound	Wound+CSSC (siTSG6)	Wound+CSSC (siCtrl)
	19	13287	13203	1456
	275	8023	7405	6378
	28	4432	6051	3002
	25	3488	22572	2487
	36	8010	8599	2560
	284	3886	3394	872
	419	17578	2992	2666
	120	2367	10104	1848
**Mean**	**150.8**	**7633.9**	**9290.0**	**2658.6**
**SD**	**144.7**	**5004.5**	**5930.2**	**1552.7**

* As shown in Fig 4B in manuscript.

p values**

No Wound vs. Wound	**	0.0014
Wound+CSSC(siCtrl) vs. Wound	*	0.0259
Wound+CSSC(siTSG6) vs. Wound+CSSC(siCtrl)	**	0.004
Wound+CSSC(siTSG6) vs. No Wound	***	0.0002
Wound+CSSC(siTSG6) vs. Wound	ns	0.4402
Wound+CSSC(siCtrl) vs. No Wound	ns	0.2458

** calculated from ordinary one-way ANOVA, unpaired, multiple comparisons. Fisher's LSD test
