# Supplementary material for: Corneal stromal stem cells reduce corneal scarring by mediating neutrophil infiltration after wounding
Source: PLoS One. 2017 Mar 3;12(3):e0171712. doi: 10.1371/journal.pone.0171712 (PMC5336198; doi:10.1371/journal.pone.0171712)
Supplement: S4 Tables — (PDF) [file pone.0171712.s004.pdf]

S4 Tables. Data supporting Fig 5

Table A Scar Area Analysis: CSSC with TSG-6 Knockdown

	Scar Area (Pixels)*	
	Control siRNA	TSG-6 siRNA
	0	27851
	0	37328
	195	84975
	205	1984
	149	7405
	0	6709
**Mean**	91.5	31908.6
**SD**	102.0	31263
**n**	6	6
**p value****	0.0279	

* Data obtained from analysis of images in S1 Fig.

** Calculated with an unpaired, one-tailed t-test.

Table B. Analysis of Fibrotic Gene Expression in wounded corneas.*

	No Wound			Wound- No CSSC			Wound CSSC(siCtrl)			Wound-CSSC(siTSG6)			p value***
	ΔΔCt**	SD	n	ΔΔCt	SD	n	ΔΔCt	SD	n	ΔΔCt	SD	n	
**Acta2**	0	0.100	3	3.500	0.140	3	0.149	0.140	3.000	0.900	0.128	3	0.0032
**Col3a1**	0	0.200	3	6.400	0.050	3	2.900	0.129	3.000	4.500	0.132	3	<0.0001
**Tnc**	0	0.171	3	1.907	0.167	3	0.550	0.131	3.000	1.336	0.128	3	0.0061

* Summarized in Fig 5B, 5B, 5C in manuscript

** These values are nomalized to No Wound samples

*** Comparing Wound-CSSC(siTSG) with Wound-CSSC(siCtrl) by ordinary one-way ANOVA, multiple comparisons, Fisher's LSD test.
