# Supplementary material for: Corneal stromal stem cells reduce corneal scarring by mediating neutrophil infiltration after wounding
Source: PLoS One. 2017 Mar 3;12(3):e0171712. doi: 10.1371/journal.pone.0171712 (PMC5336198; doi:10.1371/journal.pone.0171712)
Supplement: S5 Tables — (PDF) [file pone.0171712.s005.pdf]

S5 Tables. Supporting data for results displayed in Fig 6

Table A Scar Area Analysis - Neutropenic Mice*

	Scar Area (Pixels)	
	**Neutropenic**	**Control**
	20749	44188
	837	63680
	18512	29086
	21364	30218
**Mean**	15366	41793
**S.D.**	9763	16127
**n**	4	4
**p value****	0.0155	

* Data obtained from analysis of S2 Fig
** Calculated with an unpaired, one-tailed t-test.

Table B. Analysis of Fibrotic Gene Expression in wounded corneas.*

	No Wound			Wound -Ctrl			Wound-NP			p value***
	mRNA**	SD	n	mRNA	SD	n	mRNA	SD	n	
**Acta2**	1	0.3	3	1.8	0.26	3	1.1	0.1	3	0.011
**Col3a1**	1	0.06	3	22	1.86	3	4.8	0.11	3	<0.0001
**Tnc**	1	0.02	3	6.8	1.5	3	0.6	0.17	3	0.0001

* Summarized in Fig 6B, 6B, 6C in manuscript
** These values are nomalized to No Wound samples
*** Comparing Wound-Ctrl with Wound-NP by ordinary one-way ANOVA, multiple comparisons, Fisher's LSD test.
