# Supplementary material for: Corneal stromal stem cells reduce corneal scarring by mediating neutrophil infiltration after wounding
Source: PLoS One. 2017 Mar 3;12(3):e0171712. doi: 10.1371/journal.pone.0171712 (PMC5336198; doi:10.1371/journal.pone.0171712)
Supplement: S6 Tables — (PDF) [file pone.0171712.s006.pdf]

S6 Table. Expression levels of Kera and TNFAIP6 in Gene Array*

Gene	Keratocyte	CSSC	Fibroblast
KERA	4313	41	52
TNFAIP6	2630	456	880

* Data from Gene Array Reported Du et al. 2007 IOVS 48, 5038-5045
