# Supplementary material for: Corneal stromal stem cells reduce corneal scarring by mediating neutrophil infiltration after wounding
Source: PLoS One. 2017 Mar 3;12(3):e0171712. doi: 10.1371/journal.pone.0171712 (PMC5336198; doi:10.1371/journal.pone.0171712)
Supplement: S1 Fig — (PDF) [file pone.0171712.s007.pdf]

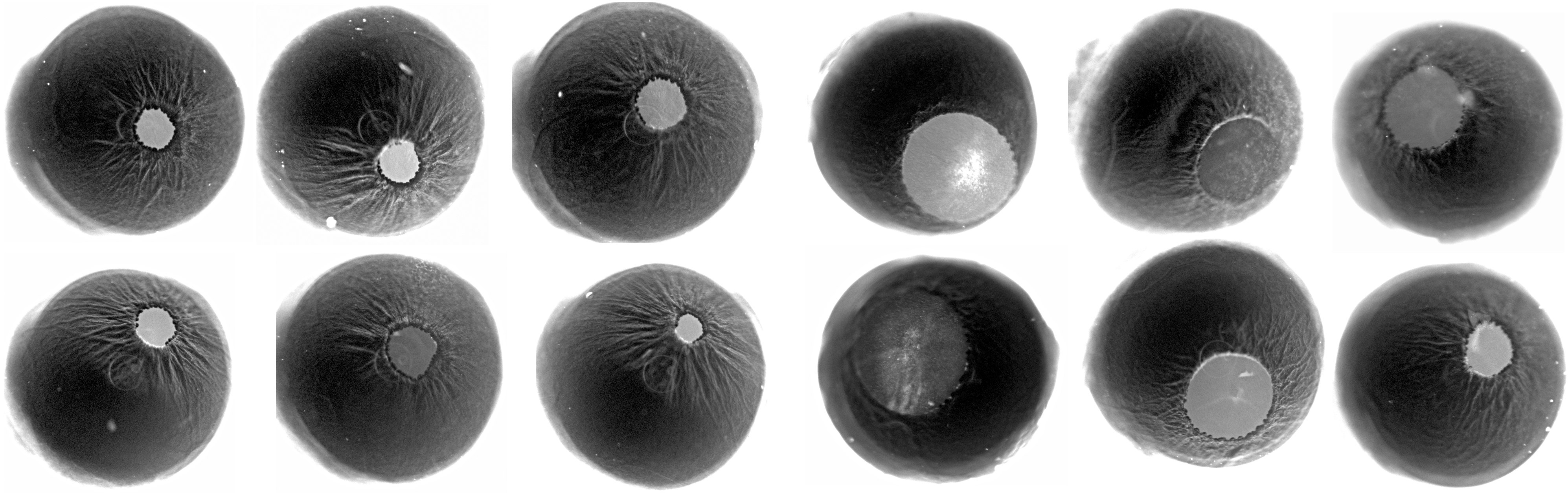

S1 Fig. Scarring in eyes treated with CSSC after TSG-6 knock-down. Fresh corneas were imaged ex vivo using indirect lighting, 14 days after wounding. Scar area was calculated from high resolution images by a trained observer in a masked fashion to obtain data in Table A in S4 Tables and Graph 5A in the manuscript. **T**, Treated with CSSC- siTSG6 RNA. **C**, Treated with CSSC-siCtrl RNA
