# Supplementary material for: Corneal stromal stem cells reduce corneal scarring by mediating neutrophil infiltration after wounding
Source: PLoS One. 2017 Mar 3;12(3):e0171712. doi: 10.1371/journal.pone.0171712 (PMC5336198; doi:10.1371/journal.pone.0171712)
Supplement: S2 Fig — (PDF) [file pone.0171712.s008.pdf]

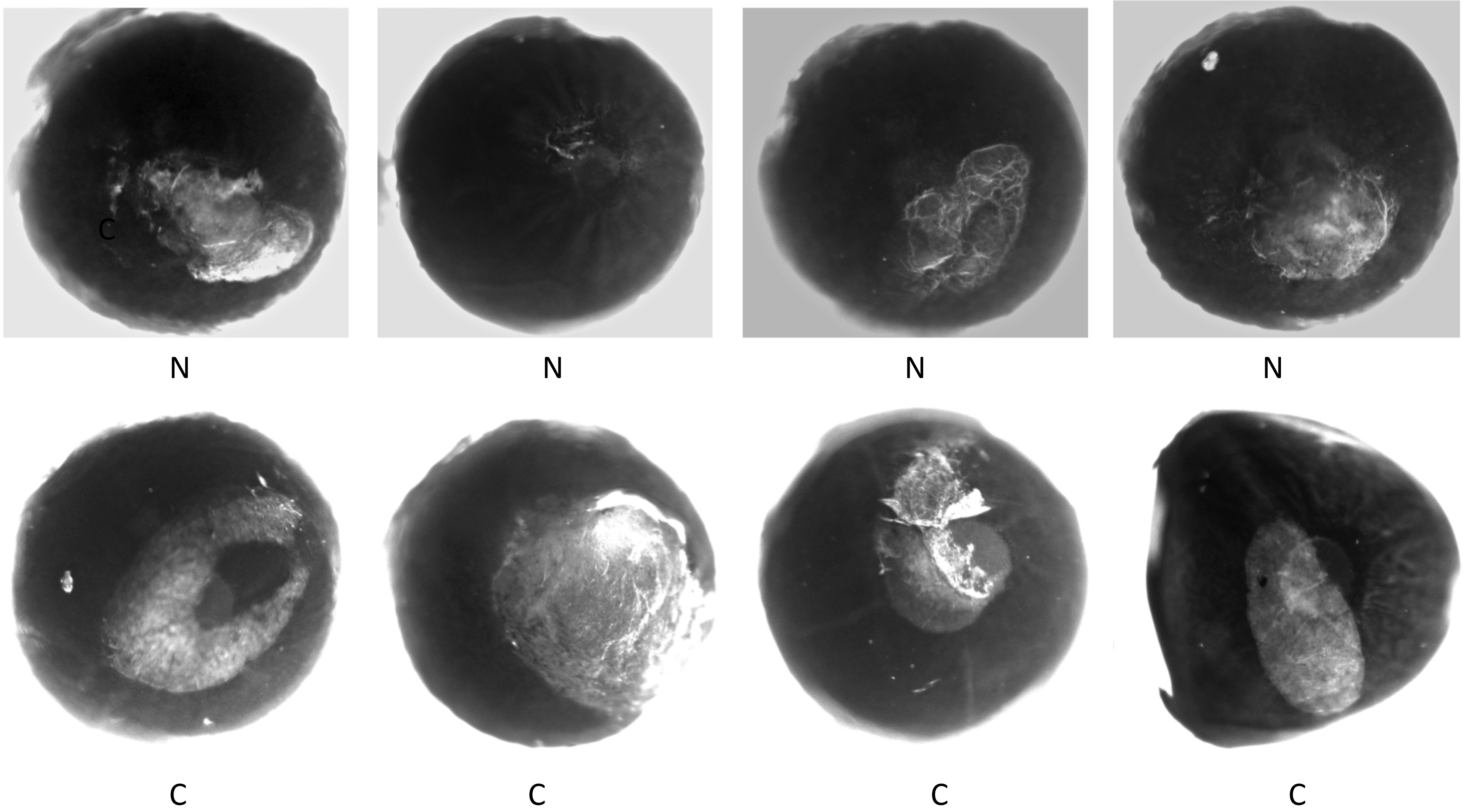

S2 Fig. Scarring in eyes of neutropenic mice. Fresh corneas were imaged ex vivo using indirect lighting 14 days after wounding. Eyes were not treated with CSSC. Scar area was calculated from high resolution images by a trained observer in a masked fashion to obtain data in Table A in S5 Tables and Fig 6A in the manuscript. **N**, Neutropenic mice **C**, Untreated mice.
